# Acute “Pseudoischemic” ECG Abnormalities after Right Pneumonectomy

**DOI:** 10.1155/2017/7872535

**Published:** 2017-01-18

**Authors:** Nada Vasic, Sanja Dimic-Janjic, Ruza Stevic, Branislava Milenkovic, Verica Djukanovic

**Affiliations:** ^1^Clinic for Pulmonary Diseases, Clinical Center of Serbia, Belgrade, Serbia; ^2^Faculty of Medicine, University of Belgrade, Belgrade, Serbia; ^3^Center for Radiology and Magnetic Resonance Imaging, Clinical Center of Serbia, Belgrade, Serbia

## Abstract

New onset of electrocardiographic (ECG) abnormalities can occur after lung surgery due to the changes in the position of structures and organs in the chest cavity. The most common heart rhythm disorder is atrial fibrillation. So-called “pseudoischemic” ECG changes that mimic classic ECG signs of acute myocardial ischemia are also often noticed. We report the case of a 68-year-old male, with no prior cardiovascular disease, who underwent extensive surgical resection for lung cancer. On a second postoperative day, clinical and electrocardiographic signs of acute myocardial ischemia occurred. According to clinical course, diagnostic procedures, and therapeutic response, we excluded acute coronary syndrome. We concluded that physical lesion of the pericardium, caused by extended pneumonectomy with resection of the pericardium, provoked the symptoms and ECG signs that mimic acute coronary syndrome. Our final diagnosis was postpericardiotomy syndrome after extended pneumonectomy and further treatment with nonsteroidal anti-inflammatory drugs (NSAIDs) was recommended. It is necessary to consider possibility that nature of ECG changes after extended pneumonectomy could be “pseudoischemic.”

## 1. Introduction

New onset of electrocardiographic (ECG) abnormalities can occur after lung surgery due to changes in the position of structures and organs in the chest cavity. As a consequence of lung resection, changes in all parts of the electrocardiogram were observed (P wave, QRS complex, ST segment, and T-wave). Heart rhythm disorders that often occur are atrial arrhythmias and most often atrial fibrillation [1]. Also so-called “pseudoischemic” ECG signs were more frequently noted in patients after extended lung resections.

## 2. Case Report

**Figure 1:**
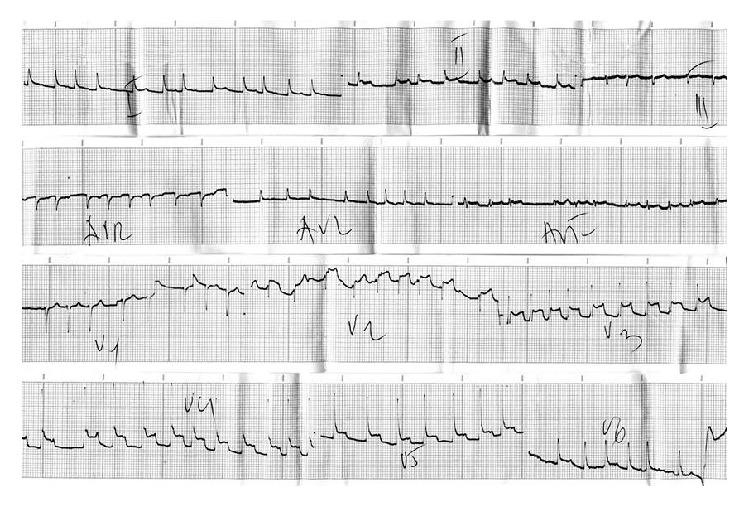
ECG shows signs of absolute arrhythmia, HR of 140/min, and ST elevation from V2 to V6, D1 and D2.

68-year-old male, with two-month symptoms (cough, hemoptysis, and body weight loss) and no prior chronic disease, was hospitalized for surgical resection of non-small-cell lung cancer (NSCLC). After standard preoperative assessment, patient was selected for surgical treatment. Clinical examination and blood analysis on admission were unremarkable. Chest X-ray revealed a tumor mass in the projection of the right hilum. On ECG, the following were observed: sinus rhythm, HR of 80/min, and no changes in ST and T. Spirometry, diffusion capacity for carbon monoxide, and arterial blood gas analysis revealed normal lung functions. Right pneumonectomy with partial resection and primary closure of the pericardium was performed, with satisfactory intraoperative and immediate postoperative course. On the second postoperative day, the patient complained of acute mid-chest pain, fatigue, and sweating. Pain had intermittent character, lasted about one hour, and spread to the back. Patient was pale and diaphoretic, with RR of 19/min and BP of 90/60 mmHg and tachyarrhythmia on ECG (supraventricular extrasystoles, 100 beats/min, and slight ST elevation in V4 to V6). Oxygen, digoxin, hydration, and nonsteroidal anti-inflammatory drugs (NSAIDs) for analgesic effects were administered. Persistence of symptoms and dynamic of the ECG findings (absolute arrhythmia, HR of 140/min, and ST elevation from V2 to V6, D1 and D2) suggested acute myocardial infarction, and patient continued treatment in the intensive coronary care unit (Figure 1).

**Figure 2:**
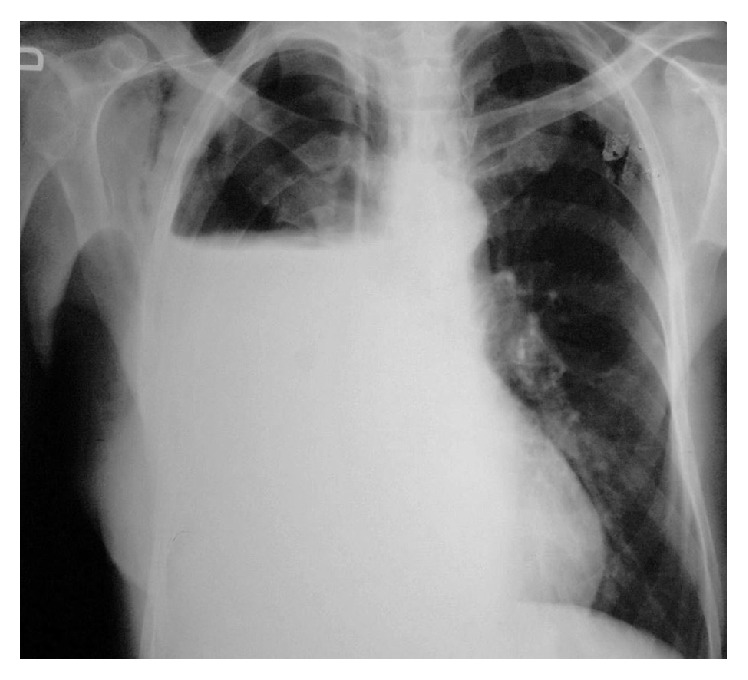
Chest X-ray shows hydroaeric level in the right hemithorax, suggesting state after pneumonectomy.

There were no wall motion abnormalities of the left ventricle or presence of pericardial effusion on transthoracic echocardiography (TEE). On ECG, the following were observed: absolute arrhythmia, HR of 140/min, and concave ST elevation in lead D2, from V2 to V6. Blood analysis revealed increased level of C-reactive protein, 68 mg/L (upper limit 5 mg/L), and troponin, 0.018 *μ*g/L (upper limit 0.04 *μ*g/L). ECG performed 72 hours after onset of symptoms showed initial normalization of ST segment and appearance of negative T waves in leads V3 to V6. Based on clinical, ECG, blood, and TEE findings, acute myocardial infarction was excluded and final diagnosis was epistenocardiac pericarditis. The treatment with antiplatelets (salicylic acid), Ca-channel antagonists* (verapamil),* and* digoxin* was successful and the patient had no symptoms; physical examination was normal, ECG showed the following: sinus rhythm (HR of 60/min) and negative T-wave from leads V2 to V6; chest X-ray corresponded with condition after right pneumonectomy (Figure 2).

We concluded that physical lesion of the pericardium, caused by extended pneumonectomy with resection of the pericardium, provoked the symptoms and ECG signs which, in its early stage, mimic acute myocardial infarction. Patient was discharged from the hospital after two weeks, without cardiovascular disorder, and further treatment with nonsteroidal anti-inflammatory drugs (NSAIDs) was recommended for one month. There were no signs of cardiac disease after one month of treatment.

## 3. Discussion

We present a case of a patient with non-NSCLC who underwent intrapericardial right pneumonectomy and had postoperative ECG signs of pseudoischemia and clinical symptoms and signs that correspond to acute myocardial infarction. Atrial fibrillation (AF) is the most common cardiac arrhythmia that occurs after the thoracic surgery with an incidence of 10% to 20% after lobectomy and even 40% after pneumonectomy. The incidence of ischemic ECG abnormalities in large series is 3.8% and myocardial infarction in 1.2% [1]. Most arrhythmias occur within the first 2 to 3 days after surgery. The incidence is higher if an intrapericardial dissection was required for resection of the tumor [2]. Risk factors for tachyarrhythmias are previous general health status (cardiovascular comorbidity, limited pulmonary reserve), type of surgical intervention (extensive resection, intrapericardial pneumonectomy, extrapleural pneumonectomy, anesthetics, and bleeding), previous oncology treatment (chest radiotherapy), older age, and procedures associated with pericardial inflammation, especially dissection around the atria [3]. Tachydysrhythmias occur more frequently after intrapericardial dissection and in patients who have postoperative interstitial or perihilar pulmonary edema [4]. AF in our patient was related to the type and extensity of lung resection (intrapericardial pneumonectomy). New-onset postoperative AF is often transient and prompt ventricular rate control is essential. Rate control therapy resolves AF in most cases in thoracic surgery. AF usually disappears during 24 hours of prescribing rate control therapy and patients are discharged with sinus rhythm [5]. We achieved and maintained the heart rate and rhythm control with previously described medical treatment approach. It could be difficult to diagnose a postoperative myocardial infarction especially if lung surgery includes pericardium. Cardiac biomarkers (troponin) have significant value in the early diagnosis, risk assessment, and timely treatment of myocardial infarction. After pneumonectomy, troponin I reached peak in the eighth postoperative hour [6]. Differentiation of new-onset postoperative chest pain, between ischemic heart disease and postthoracotomy pain, could be difficult. Patients with only marginally increased cTnI level after intrapericardial resections or pneumonectomy should remain in the intensive care unit and should be monitored carefully [6].

Significant ST elevation may occur if lavage of chest cavity is performed. It tends to occur during irrigation, stops with cessation, and is associated with the TEE findings and other hemodynamic changes that are suggestive of myocardial infarction. The incidence of preoperative MI was low (0.13%) in patients with no previous cardiac history to moderate (2.8% to 17%) in patients with a prior history of infarction. The authors recommend continuous monitoring for at least 3 days, certainly in high-risk patients, as postoperative MI was associated with mortality as high as 32% to 70% [7].

This could represent the electrophysiological changes that are not ischemic but associated with focal myocardial heat-related surface electrolyte changes. Treatment is not necessary, except in hemodynamic instability leading to or associated with an abnormal wall motion by TEE [8]. “Pseudoischemic” ST segment elevation in ECG is described in a few patients that underwent thoracotomy. Chhabra et al. described new changes in ST segment and T-wave (ST-T), often found in the early postoperative period. The most common disorders were ST-depressions observed in 35% patients. In our case the diffuse ST elevation was the initial change in the ST-T segment. Flattened T-wave and/or the T-wave inversion in the lower leads and lateral precordial leads also indicated the new transient abnormality in acute postoperative course in 7.7% of patients [9]. The dynamics of ECG changes may have timed relationship with the changes concerning mediastinal shift postoperatively.

Studies have shown that surgical factors (irritation of the atria per se, chronic inflammation, and ageing of atria), direct surgical trauma to autonomic nervous system, and postthoracotomy pain can interdependently influence the onset of the arrhythmia [10]. ECG changes that mimic ischemia may be the consequence of previous lung resection as well [11].

Thoracic surgery is a well-known cause of recurrent pericarditis. Pericarditis is typically identified by recurrences of chest pain and/or pericardial rub and is monitored by ECG or pericardial inflammation shown by echocardiography [12]. ECG changes suggestive of recurrent acute pericarditis were present in 41% of patients with this condition and echocardiographic evidence was present in 86% of those patients in a study by Fowler and Harbin III [13]. Electrocardiographic changes included ST segment elevation with subsequent ST normalization [14]. Nonsteroidal inflammatory agents have traditionally been used as first-line monotherapy for uncomplicated, isolated attacks of acute pericarditis. Steroids have been the most widely used agents for suppression of recurrent pericarditis [14]. The same treatment was prescribed to our patient. We made the diagnosis of postpneumonectomy pericarditis in our patient according to clinical, ECG, and TEE signs and treatment response to NSAID. Acute coronary syndrome was excluded by cardiac biomarkers and disease outcome.

Elevation of cardiac troponin I (cTnI) correlates with the fact that intrapericardial pneumonectomy predisposes pericardial trauma. Troponin may be increased by primary or secondary ischemic cardiac trauma. Numerous clinical studies have shown that interventions that include myocardial revascularization may be accompanied by an increase in troponin levels [15].

Postpericardiotomy syndrome occurs in days or months after a cardiac or pericardial trauma with characteristics similar to postinfarct syndrome. Both syndromes are variations of a common immune-pathological process. In contrast to the postinfarct syndrome, postpericardiotomy injury syndrome leads to acute injury and has a more pronounced immune response, in the sense of stronger anti-heart antibody response (anti-sarcolemmal and anti-fibrillar antibodies), probably related to more extensive release of antigenic material [12, 15].

## 4. Conclusion

Identifying and understanding the changes in ECG findings after lung resection is very important, particularly for exclusion of other acute cardiopulmonary events, mainly myocardial infarction and pulmonary thromboembolism in timely manner. Postpneumonectomy ECG changes have certain dynamics and may reflect strong connection with postoperative changes in the anatomy of the chest cavity. Differential diagnosis of pain caused by cardiac ischemia and early postoperative pain period is often difficult. Even a slight increase in cardiac biomarkers after intrapericardial pneumonectomy requires close clinical assessment and follow-up for early differential diagnosis between acute myocardial infarction and pseudoischemic changes due to extended intrapericardial lung resection.
